# Effectiveness of emulsion bathing in adult patients with atopic dermatitis

**DOI:** 10.5415/apallergy.0000000000000186

**Published:** 2025-03-17

**Authors:** Duy Le Pham, Nguyet Doan Lam Nguyen, Quoc Quang Luu

**Affiliations:** 1 Department of Physiology, Pathophysiology and Immunology, Faculty of Medicine, University of Medicine and Pharmacy at Ho Chi Minh City, Ho Chi Minh City, Vietnam; 2 Unit of Allergy and Clinical Immunology, University Medical Center Ho Chi Minh City, Ho Chi Minh City, Vietnam; 3 Department of Dermatology, Allergy and Clinical Immunology, Thong Nhat Hospital, Ho Chi Minh City, Vietnam; 4 Department of Oral & Maxillofacial Surgery, Loma Linda University School of Dentistry, Loma Linda, CA, USA

**Keywords:** Atopic dermatitis, bath oil, moisturizing, Stratum corneum hydration, transepidermal water loss

## Abstract

**Background:**

Emulsion bathing has been commonly advised in the management of atopic dermatitis (AD) to maintain skin hydration and reduce the negative effects of soaps on the skin barrier. However, there is limited scientific evidence from randomized controlled trials to support their effectiveness.

**Objectives:**

We aimed to compare the moisturizing effectiveness of 2 regimens: emulsion bathing combined with moisturization and moisturization after bathing with tap water only, in adults with AD.

**Method:**

Thirty-nine adult patients with AD (aged 20–63 years) were recruited. The right and left forearms of each subject were randomly assigned to be immersed and washed for 3 minutes with either moisturizing bath oil (dual-therapy group) or tap water alone (monotherapy group), followed by air drying for 30 minutes. Subsequently, a moisturizer was applied to the tested skin areas. Stratum corneum hydration (SCH) and transepidermal water loss (TEWL) were measured using a GP Skin Pro device (Gpower, South Korea) at five time points: before washing (baseline), after washing and air drying for 30 min (postwash), and after moisturization at 0, 30 and 60 minutes.

**Results:**

At the postwash time point, mean SCH levels in both groups decreased significantly; however, the mean SCH level of the dual-therapy group was significantly higher than that of the monotherapy group (20.9 ± 8.4 a.u. vs 14.4 ± 9.96 a.u., *P =* 0.006). Additionally, the mean TEWL level of the dual-therapy group was significantly lower than that of the monotherapy group (6.73 ± 2.7 g/m^2^/h vs 8.53 ± 3.1 g/m^2^/h, *P =* 0.009). After moisturizer application, both study groups demonstrated significant increases in SCH and decreases in TEWL levels. However, no significant differences in SCH and TEWL levels were observed between the 2 groups after moisturization at 0, 30, and 60 minutes.

**Conclusion:**

Emulsion bathing helped prevent epidermal damage induced by bathing. However, the combination of emulsion bathing and moisturization did not show superiority over moisturization alone in improving skin hydration after bathing.

## 1. Introduction

Dry skin is an early manifestation of epidermal barrier damage, characterized by reduced water retention in the stratum corneum, a hallmark of many inflammatory skin diseases, most commonly atopic dermatitis (AD) [1]. Moisturizer application is a simple, easy-to-apply, and effective approach to alleviating dry skin symptoms. In addition to the traditional method of moisturizer application, emulsion bathing has been recommended as a method to maintain skin hydration and water retention, reduce transepidermal water loss (TEWL), and avoid the negative effects of detergents and cleansers found in conventional body washes.

The European Academy of Dermatology and Venereology and the National Institute for Health and Care Excellence guidelines for diagnosing and managing AD recommend incorporating moisturizing bathing into daily bathing routines to optimize moisturizing efficacy [2, 3]. Several studies have investigated the effectiveness of oil bathing or emulsion bathing in children with or without AD [4]. However, there are limited randomized controlled clinical trials in adults with AD. Therefore, this study was conducted to evaluate the moisturizing effectiveness of emulsion bathing in combination with moisturizer application in skincare regimens for adult patients with AD.

## 2. Study subjects and methods

### 2.1. Study subjects

A randomized controlled clinical trial was performed with 39 patients diagnosed with AD at the Allergy - Clinical Immunology Clinic, University Medical Center Ho Chi Minh City, between November 2023 and June 2024. AD was diagnosed based on the Hanifin and Rajka criteria. Subjects were excluded from the study if they: (1) had a history of hypersensitivity reactions to topical medications or cosmetics or developed hypersensitivity reactions during the study; (2) applied moisturizer, or sunscreen on the forearm skin within 12 hours before the study, (3) or wished to withdraw from the study at any point. All patients provided written informed consent at the time of recruitment.

The study was approved by the Biomedical Research Ethics Committee of the University of Medicine and Pharmacy at Ho Chi Minh City (IRB number: IRB-VN01002/IORG0008603/FWA00023448). Prof Dung Van Do, MD, PhD is the president of this Ethics Committee.

### 2.2. Study procedure

Two volar forearms of each participant were randomly assigned to either emulsion bathing (dual-therapy group) or tap water bathing (monotherapy group). First, each forearm was washed with tap water to remove residual sweat or sebum, then patted with a paper towel and air-dried for 30 minutes. Baseline stratum corneum hydration (SCH) and TEWL levels were measured. An emulsion bathing oil was evenly applied to one volar forearm (dual-therapy group) for 1 minute, while the other forearm was left untreated (monotherapy group). Subsequently, each forearm was immersed in separate basins of tap water for 3 minutes, then patted dry and air-dried for 30 minutes in a room with controlled air-conditioning with a temperature of 25 ± 2°C and relative humidity of 60% to 70%. Postwash SCH and TEWL levels were measured. A moisturizer was then applied to both forearms. SCH and TEWL were measured immediately (T0), and at 30 minutes (T30) and 60 minutes (T60) after moisturizer application. At each time point, measurements were performed in triplicates to obtain the average SCH and TEWL levels.

The bath emulsion contains urea as a humectant, and fatty acids as emollients, including sunflower seeds, corn, sesame, olive oil, macadamia oil, blackcurrant seed oil, Cardiospermum halicacabum flower extract, and sunflower seed oil. The bath emulsion is free from paraffin, petroleum derivatives, fragrances, color additives, and parabens.

The moisturizer contains water, hydrogenated polydecene, Simmondsia chinensis (jojoba) seed oil extract, butylene glycol, cyclopentasiloxane, glycerin, behenyl alcohol, glyceryl stearate, PEG 60, glyceryl isostearate, cetyl alcohol, pentylene glycol, trideceth 12, sodium lauroyl lactylate, sorbitan stearate, beeswax, dimethicone, peg 32, peg 6, hydroxypropyl bispalmitamide mea, phenoxyethanol carbomer, ethylhexylglycerin, cholesterol, linoleic acid, tocopherol, xanthan gum, sodium hyaluronate, sodium hydroxide.

### 2.3. Statistical analysis

Statistical analyses were performed using Stata 14 (StataCorp LLC, College Station, TX, USA). The confidence interval was set at 95%, and a *P* value of ≤0.05 was considered statistically significant. Differences in mean values between the 2 groups were compared using the Student *t* test for normal distributions or the Mann–Whitney *U* test for nonnormal distributions. Graphs were created using GraphPad Prism 10 (GraphPad Software Inc., Boston, MA, USA).

## 3. Results

### 3.1. Clinical characteristics of the study subjects

The study included 39 participants with a mean age of 27.8 ± 9.44 years, ranging from 20 to 63 years. The clinical characteristics of the participants are presented in Table 1.

**Table 1.** Clinical characteristics of the study subjects

Clinical feature	n	Prevalence (%)
Gender		
Male	10	25.6%
Female	29	74.4%
SCORAD		
Mild (0–25)	26	66.7%
Moderate (25–50)	7	17.9%
Severe (>50)	6	15.4%
AD treatment		
** **Moisturizer application	18	46.2%
** **Local anti-inflammation	17	43.6%
** **Systemic anti-inflammation	2	5.1%

AD, atopic dermatitis; SCORAD, scoring atopic dermatitis.

### 3.2. Comparison of the SCH levels between the 2 regimens

**Figure 1.**
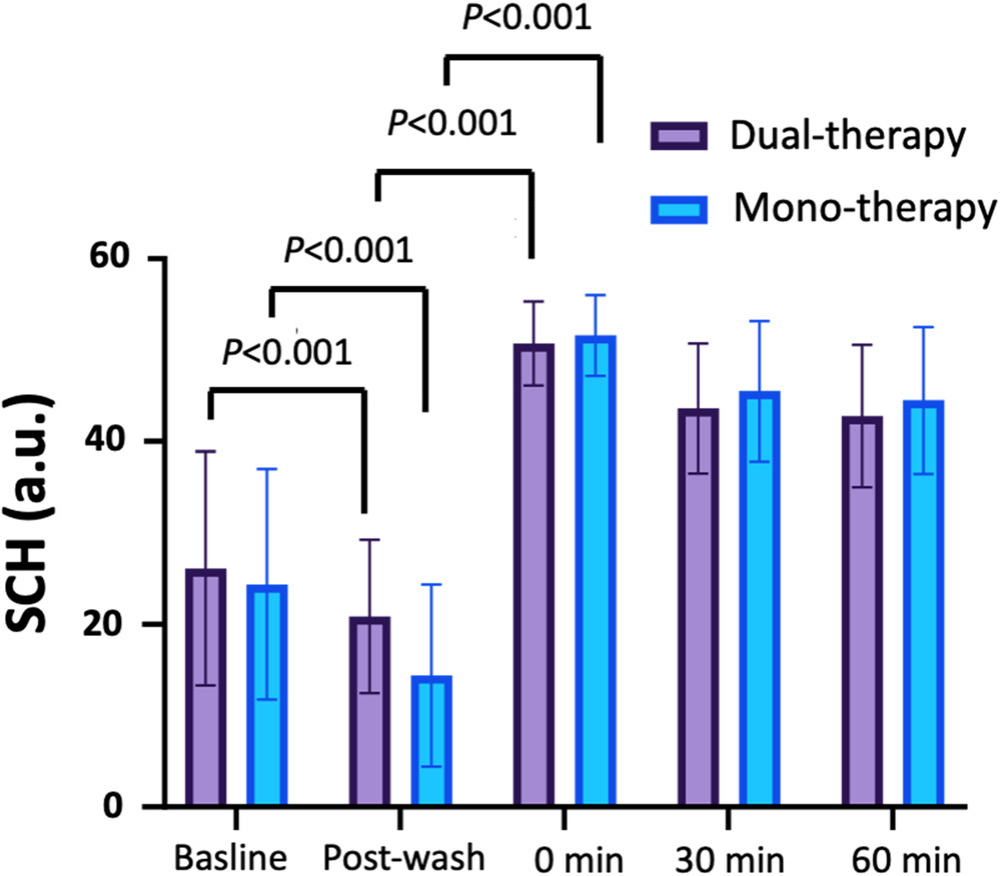
Mean stratum corneum hydration (SCH) levels of the dual-therapy group and monotherapy group at each time point. *P* values were obtained by the Mann–Whitney *U* test.

The mean SCH levels at baseline of the entire study population were 25.3 ± 12.2 a.u. The mean SCH levels of each group at different time points are shown in Table 2 and Figure 1.

**Table 2.** Comparison of SCH levels between the dual-therapy group and monotherapy group at different time points

SCH (a.u.)	Dual-therapy group			Monotherapy group			*P* value
	Median	IQR	Mean	Median	IQR	Mean	
Baseline	26.33	14.7–35.7	26.1 ± 12.8	22.67	14.7–35	24.4 ± 12.6	0.522
Postwash	20.33	15–26	20.9 ± 8.4	15.33	4–20.33	14.4 ± 9.96	**0.006**
0 min	51.33	47.3–56	50.7 ± 4.6	52	48.7–54.7	51.6 ± 4.42	0.421
30 min	43.33	40–50	43.6 ± 7.1	47	41–49.7	45.5 ± 7.66	0.168
60 min	43.67	37.3–50	42.8 ± 7.8	45.33	37.7–51.7	44.5 ± 8.05	0.320

*P* values were obtained by the Mann–Whitney *U* test. Bold values indicate statistical significance.

IQR, interquartile range; SCH, stratum corneum hydration.

After washing, the postwash SCH levels were significantly decreased compared with the baseline SCH levels in both dual-therapy group and monotherapy group (*P* < 0.001 for both groups, Fig. 1). However, the postwash SCH levels of the dual-therapy group (20.9 ± 8.4 a.u.) was significantly higher than in the monotherapy group (14.4 ± 9.96 a.u, *P =* 0.006; Table 2). At 0 minutes after moisturizer application, the SCH levels in both groups increased significantly compared with the postwash levels (*P* < 0.001 for both groups; Fig. 1). There were no statistically significant differences in SCH levels between the dual-therapy group and monotherapy group after moisturizer application at 30 minutes and 60 minutes after moisturizer application.

### 3.3. Comparison of the TEWL levels between the 2 regimens

**Figure 2.**
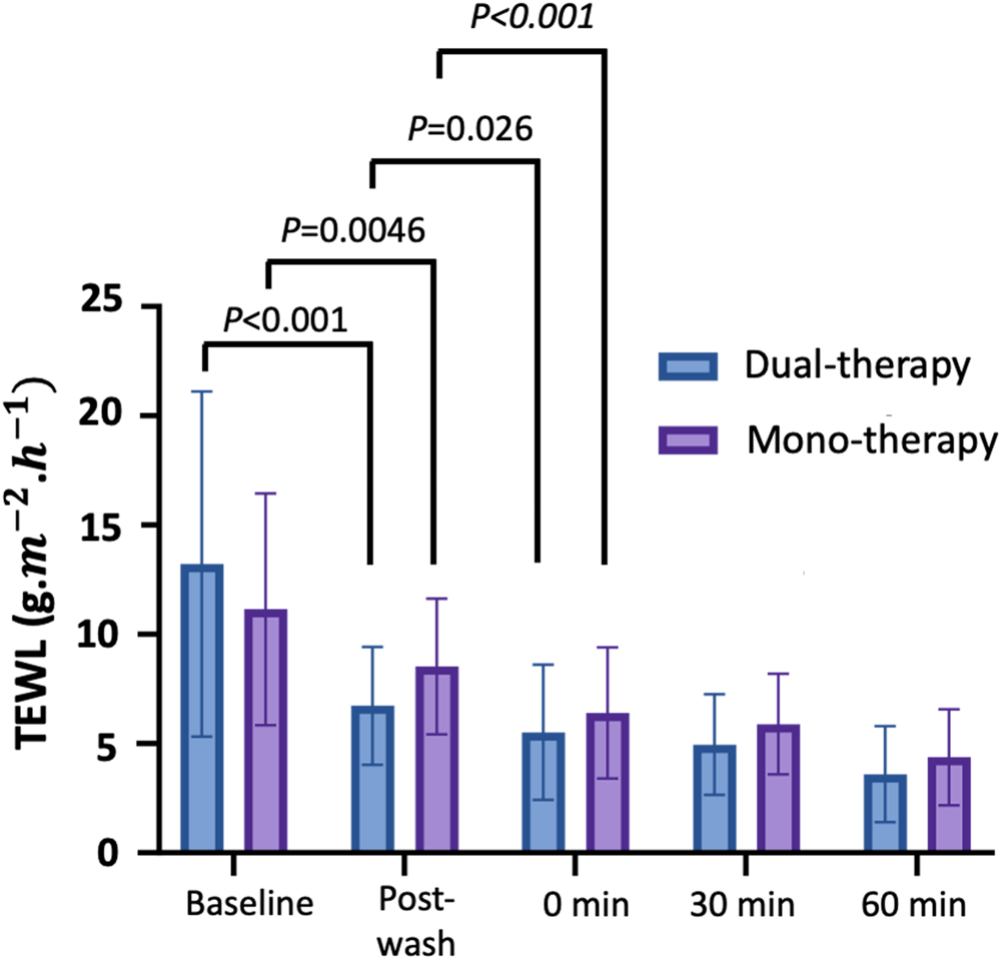
Mean transepidermal water loss (TEWL) levels of the dual-therapy group and monotherapy group at each time point. *P* values were obtained by the Mann–Whitney *U* test.

The mean TEWL levels at the baseline of the entire study population were 12.18 ± 5.97 g/m^2^/h. The mean TEWL levels of each group at different time points are presented in Table 3 and Figure 2.

**Table 3.** Comparison of TEWL levels between dual-therapy group and monotherapy group at different time points

TEWL (g/m^2^/h)	Dual-therapy group			Monotherapy group			*P* value
	Median	IQR	Median	IQR	Median	IQR	
Baseline	11.3	8.5–17.1	13.22 ± 7.9	10	7.5–13.3	11.15 ± 5.3	0.257
Postwash	7	4.4–8.7	6.73 ± 2.7	8.5	5.7–10.6	8.53 ± 3.1	**0.009**
0 min	5.1	2.97–7.7	5.52 ± 3.1	6.57	3.6–8.1	6.41 ± 3.0	0.238
30 min	4.9	3.6–6.1	4.95 ± 2.3	5.7	4.0–7.3	5.89 ± 2.3	0.089
60 min	2.9	1.7–5.1	3.60 ± 2.2	4.0	2.97–6.4	4.38 ± 2.2	0.059

*P* values were obtained by the Mann–Whitney *U* test. Bold values indicate statistical significance.

IQR, interquartile range; TEWL, transepidermal water loss.

After washing, the postwash TEWL levels were significantly decreased compared with the baseline TEWL levels in both the dual-therapy group (*P* < 0.001) and monotherapy group (*P =* 0.0046; Fig. 2). However, the postwash TEWL levels of the dual-therapy group (6.73 ± 2.7 g/m^2^/h) were significantly lower than those in the monotherapy group (8.53 ± 3.1 g/m^2^/h^1^, *P =* 0.009; Table 3). At 0 minutes after moisturizer application, the TEWL levels were further decreased significantly in both the dual-therapy group (*P =* 0.026) and monotherapy group (*P* < 0.001, Fig. 2). There were no statistically significant differences in TEWL levels between the dual-therapy group and monotherapy group after moisturizer application at 30 minutes and 60 minutes after moisturizer application.

## 4. Discussion

The present study investigated the moisturizing effectiveness of emulsion bathing in adult patients with AD, a topic that has not been well studied. We found that the bathing process significantly compromised skin hydration, but this effect could be minimized by emulsion bathing. In addition, moisturizer application after bathing significantly restored skin hydration, which was maintained for at least 60 minutes. Nevertheless, emulsion bathing combined with moisturization did not increase the moisturizing effectiveness compared with moisturization alone in this short-term observation.

Washing is beneficial in eliminating skin debris as well as harmful microorganisms on the skin, which is necessary for the management of AD. However, it has been found to negatively affect the epidermal barrier [5]. Our study also showed that the SCH values measured at 30 minutes after bathing with either water only or bathing emulsion significantly decreased, reflecting a decline in epidermal hydration induced by the bathing process. The finding was in line with the previous reports [5]. These findings suggest that washing could compromise the skin barrier and eliminate the natural moisture of stratum corneum, which does not effectively recover by itself at least 30 minutes after washing and air drying. The period after washing and before moisturization could be considered a “window time” when the eczema-affected skin is sensitive and prone to damage induced by environmental factors such as low humidity and temperature [6].

Nevertheless, the forearms that were washed with a bath emulsion had significantly higher postwash SCH levels and lower TEWL levels compared with those washed with only tap water. This finding indicates that bath emulsion could help reduce skin dehydration after washing by either adding moisture to the skin or minimizing the loss of epidermal water content induced by evaporation. Bath emulsion may contain the humectants that help the epidermis to absorb water from the surroundings, thereby increasing the moisture of the epidermis. In addition, occlusives in the bath emulsion produce a hydrophobic barrier over the epidermis, thereby preventing the loss of epidermal water content by evaporation. As a result, bathing with an emulsion could reduce the dehydration of the skin observed immediately after washing.

In the PreventADALL study [7] oil baths 4 times weekly from 2 weeks to 8 months in neonates were found to damage the skin barrier and increase in TEWL levels in infants at 3 and 6 months. However, the effect was no longer observed at 12 months of age. Our study found a better hydration effect of emulsion bathing compared with water alone in adults with AD, which aligns with a previous study in subjects aged 0.5 to 85 years (mean 40) [8]. These findings suggest that bathing emulsion could have different effects on children and adults. The positive effectiveness of emulsion bathing could only be observed in adult individuals and in those with eczematous skin.

In addition, the ingredients of the bathing emulsion could also contribute to hydration effectiveness. While the bath oil used in PreventADALL study contained only occlusive (paraffinum liquidum), the bathing emulsion used in our study contained not only occlusives but also emollients and humectants. Humectants help increase the moisture of the epidermis while emollients help to restore the integrity of the epidermal barrier [9]. Consequently, the combination of the 3 main hydration components (including humectant, emollient, and occlusive) in the bathing emulsion could be important for achieving better hydration effects on the skin.

Our previous study found that applying moisturizers immediately or 30 minutes after washing did not result in significantly different moisturizing effectiveness over 3 hours [10]. However, the eczema-affected skin after washing and for 30 minutes (“window time”) was dry and could be prone to further damage. The present study showed better postwash SCH and TEWL levels in the dual-therapy group compared with the monotherapy group. This finding suggests that emulsion bathing could minimize skin dehydration during the window time after washing, which could be beneficial for eczema-affected skin during the period of waiting for the moisturizer application.

After applying the moisturizer, SCH levels increased, and TEWL levels decreased dramatically in both the dual-therapy and monotherapy groups. The moisturizing effectiveness occurred immediately after moisturization, which was maintained for 60 minutes during the study period. Our previous study also found that moisturization after bathing significantly increased SCH and decreased TEWL levels, maintaining skin hydration for 180 minutes after applying moisturizers [10]. Chiang and Eichenfield [11] reported that bathing and moisturization increased skin hydration significantly within 5 to 10 minutes, which then decreased significantly, while moisturization alone increased and maintained skin hydration during 120 minutes. The differences in findings may be due to the differences in the duration of bathing (3 minutes in our study and 10 minutes in the previous study), and a longer time of bathing could induce more severe damage to the skin barrier [5]. In addition, the type of moisturizers used in the studies could also influence the hydration effectiveness after application.

Nevertheless, there was no significant difference in SCH and TEWL levels between dual-therapy and monotherapy groups, indicating that the combination of emulsion bathing and moisturization did not have superior hydration effectiveness compared with moisturization alone. It is possible that skin hydration reached a saturation level after moisturizer application, which could have masked the hydration effectiveness of emulsion bathing.

The strength of our study lies in conducting a clinical trial in adult patients with current AD, a population that has not been studied well. However, a considerable limitation is that we did not investigate the long-term effectiveness of emulsion bathing in improving skin hydration as well as managing AD symptoms.

In conclusion, in adults with AD, the bathing process significantly compromised the skin barrier and reduced skin hydration, but this effect was minimized by emulsion bathing. However, the combination of emulsion bathing and moisturizer application did not show superiority over moisturizer application alone in this short-term observation.

## Conflicts of interest

The authors have no financial conflicts of interest.

## Author contributions

DLP joined in study design, patient recruitment, data analysis and interpretation, manuscript preparation, supervising all the steps, and approving the manuscript for submission. NDLN joined in study design, patient recruitment, data analysis and interpretation, and manuscript preparation. QQL joined in manuscript preparation and submission.
